# Global Variation of Nutritional Status in Children Undergoing Chronic Peritoneal Dialysis: A Longitudinal Study of the International Pediatric Peritoneal Dialysis Network

**DOI:** 10.1038/s41598-018-36975-z

**Published:** 2019-03-20

**Authors:** Franz Schaefer, Laura Benner, Dagmara Borzych-Dużałka, Joshua Zaritsky, Hong Xu, Lesley Rees, Zenaida L. Antonio, Erkin Serdaroglu, Nakysa Hooman, Hiren Patel, Lale Sever, Karel Vondrak, Joseph Flynn, Anabella Rébori, William Wong, Tuula Hölttä, Zeynep Yuruk Yildirim, Bruno Ranchin, Ryszard Grenda, Sara Testa, Dorota Drożdz, Attila J. Szabo, Loai Eid, Biswanath Basu, Renata Vitkevic, Cynthia Wong, Stephen J. Pottoore, Dominik Müller, Ruhan Dusunsel, Claudia Gonzalez Celedon, Marc Fila, Lisa Sartz, Anja Sander, Bradley A. Warady, International Pediatric Peritoneal Dialysis Network (IPPN) Registry, M. Adragna, P. A. Coccia, A. Suarez, P. G. Valles, R. Salim, L. Alconcher, K. Arbeiter, K. van Hoeck, V. Koch, J. Feber, E. Harvey, C. White, M. Valenzuela, J. Villagra, F. Cano, M. A. Contreras, A. Vogel, P. Zambrano, P. Hevia, M. C. Chiu, Jie Ding, J. J. Vanegas, L. M. Higuita, G. Roussey, T. Ulinski, S. Krid, M. Fischbach, J. Harambat, Ch. Samaille, R. Büscher, J. Oh, L. Pape, U. John, G. Klaus, H. Billing, C. Stafanidis, F. Papachristou, A. Bagga, M. Kanitkar, R. Sinha, S. Sethi, E. Verrina, E. Vidal, G. Leozappa, D. Landau, I. S. Ha, K. H. Paik, A. Bilal, E. Sahpazova, Y. N. Lim, L. Sanchez Barbosa, J. W. Groothoff, Y. Konijenberg, Y. Silva, M. Al Ryami, R. Loza Munarriz, B. Leszczynska, M. Szczepanska, O. Brumariu, J. Kari, D. Kruscic, H. K. Yap, G. Ariceta, M. Aguirre, F. Santos, B. Niwinska-Faryna, A. Bayazit, C. A. S. Bakkaloglu, S. Bakkaloglu, I. Bilge, O. Yavascan, S. Mir, Eva Simkova, M. Christian, L. Greenbaum, A. Neu, D. Askenazi, A. Al-Akash, S. Swartz, P. Brophy, M. Rheault, M. Pradhan

**Affiliations:** 1 Center for Pediatrics and Adolescent Medicine, Heidelberg, Germany; 2 Institute of Medical Biometry and Informatics, University of Heidelberg, Heidelberg, Germany; 3 Medical University of Gdansk, Department of Pediatrics, Nephrology and Hypertension, Gdańsk, Poland; 4 Nemours/A.I. duPont Hospital for Children, Wilmington, DE USA; 5 Children’s Hospital of Fundan University, Shanghai, China; 6 Great Ormond Street Hospital, London, United Kingdom; 7 Department of Pediatric Nephrology, National Kidney and Transplant Institute, Quezon City, Philippines; 8 Dr. Behcet Uz Children Research and Educational Hospital, Izmir, Turkey; 9 Iran University of Medical Sciences, Tehran, Iran; 10 Nationwide Children’s Hospital, Columbus, OH USA; 11 Carrahpasa School of Medicine, Istanbul, Turkey; 12 University Hospital Motol, Prague, Czech Republic; 13 Seattle Children’s Hospital, Seattle, WA USA; 14 S.E.N.N.I.A.D, Montevideo, Uruguay; 15 Department of Nephrology, Starship Children’s Hospital, Auckland, New Zealand; 16 HUCH Hospital for Children and Adolescents, Helsinki, Finland; 17 Istanbul University, Istanbul Faculty of Medicine, Istanbul, Turkey; 18 Service de Néphrologie Pédiatrique, Hôpital Femme Mère Enfant, Hospices Civils de Lyon, Lyon, France; 19 Children’s Memorial Health Institute, Warsaw, Poland; 20 Pediatric nephrology, Dialysis and Transplantation Unit Fondazione IRCCS Ca’ Granda Osp. Maggiore Policlinico, Milan, Italy; 21 Jagellonian University Medical College, Kraków, Poland; 22 Semmelweis University, Budapest, Hungary; 23 Dubai Hospital, Dubai, United Arab Emirates; 24 NRS Medical College & Hospital, Kolkata, India; 25 Children Hospital, affiliate of Vilnius University Hospital Santaros Klinikos, Vilnius, Lithuania; 26 Lucile Packard Children’s Hospital at Stanford, Palo Alto, USA; 27 Children’s Medical Center Dallas, Dallas, Tx USA; 28 Department of Pediatric Gastroenterology, Nephrology and Metabolism, Charité, Berlin, Germany; 29 Erciyes University, Kayseri, Turkey; 30 Hospital Sotero del Rio, Santiago, Chile; 31 CHU Arnaud de Villeneuve, Université de Montpellier, Montpellier, France; 32 Barnkliniken, Lund, Sweden; 33 Children’s Mercy Hospital, Kansas City, MO USA; 34 Hospital de Pediatria Garrahan, Buenos Aires, Argentina; 35 Hospital Italiano de Buenos Aires, Buenos Aires, Argentina; 36 Hospital de Ninos Sor. Maria Ludovica La Plata, La Plata, Argentina; 37 Hospital Pediatrico Humberto Notti, Mendoza, Argentina; 38 R.S.A., Salta, Argentina; 39 Hospital Interzonal General, Bahia Blanca, Argentina; 40 Medical University Vienna, Vienna, Austria; 41 University Hospital Antwerp, Edegem, Belgium; 42 Instituto da CrianÇa - Hospital das Clinicas FMUSP, Sao Paulo, Brazil; 43 Children’s Hospital of Eastern Ontario, Ottawa, Canada; 44 Hospital for Sick Children, Toronto, Canada; 45 BC Children’s Hospital, Vancouver, Chile; 46 Hospital Guillermo Grant Benavente, Concepcion, Chile; 47 Hospital Base, Osorno, Chile; 48 Hospital Luis Calvo Mackenna, Santiago, Chile; 49 Roberto del Rio Hospital, Santiago, Chile; 50 Pontivicia Universidad Catolica de Chile, Santiago, Chile; 51 Hospital Dr. Gonzales Cortes, Santiago, Chile; 52 Hospital San Juan de Dios, Santiago, Chile; 53 Department of Pediatric & Adolescent Medicine, Hong Kong, China; 54 Peking First Hospital, Beijing, China; 55 Instituto del Rinon, Medellin, Colombia; 56 Baxter Servicio al Cliente Colombia, Medellin, Colombia; 57 CHU Nantes, Nantes, France; 58 Armand Trousseau Hospital, Paris, France; 59 Hopital Necker_Enfants Malades, Paris, France; 60 Children’s Dialysis Center, Strasbourg, France; 61 Hopital de Enfants, Bordeaux, France; 62 Hopital Jeanne de Flandre, Lille, France; 63 Children’s Hospital Essen, Essen, Germany; 64 University Medical Center, Hamburg, Germany; 65 Medical School, Hannover, Germany; 66 Kidney Center for Children and Adolescents, Jena, Germany; 67 KfH Kidney Center, Marburg, Germany; 68 Children’s University Hospital, TÜbingen, Germany; 69 A&P Kyriakou Children’s Hospital, Athens, Greece; 70 Aristoteles University, Thessaloniki, Greece; 71 All India Institute of Medical Sciences, New Delhi, India; 72 Armed Forces Medical College, Pune, India; 73 Institute of Child Health, Kolkata, India; 74 The Medicity, Gurgaon, India; 75 G. Gaslini Institute, Genova, Italy; 76 Pediatric Nephrology, Dialysis and Transplant Unit, Padova, Italy; 77 Department of Nefrologia-Urologia, Rome, Italy; 78 Soroka Medical Center, Beer-Sheva, Israel; 79 Seoul National University Children’s Hospital, Seoul, Korea; 80 Samsung Medical Center, Seoul, South Korea; 81 Rafik Hari University Hospital, Beirut, Lebanon; 82 Pediatric Clinic, Skopje, Macedonia; 83 Kuala Lumpur Hospital, Kuala Lumpur, Malaysia; 84 Pediatric Hospital Medical Center SXXI, Cuahutemoc, Mexico; 85 Academic Medical Center, Amsterdam, The Netherlands; 86 Wilhelmina Children’s Hospital, Utrecht, The Netherlands; 87 Hospital Infantil de Nicaragua, Managua, Nicaragua; 88 Royal Hospital, Muscat, Oman; 89 Cayetano Heredia Hospital, Lima, Peru; 90 Public Pediatric Teaching Hospital, Warsaw, Poland; 91 Dialysis Division for Children, Zabrze, Poland; 92 St. Maria Children’s Hospital, Iasi, Romania; 93 King Abdul Aziz University Hospital, Jeddah, Saudi Arabia; 94 University Children’s Hospital, Belgrade, Serbia; 95 Shaw-NKF-NUH Children’s Kidney Center, Singapore, Singapore; 96 University Hospital Materno-Infantil Vall d’Hebron, Barcelona, Spain; 97 Hospital de Cruces, Baracaldo, Spain; 98 Hospital Universitario Central de Asturias, Oviedo, Spain; 99 Karolinska University Hospital, Stokholm, Sweden; 100 Cukrova University, Adana, Turkey; 101 Hacettepe University, Ankara, Turkey; 102 Gazi University, Ankara, Turkey; 103 Department of Pediatric Nephrology, Capa-Istambul, Istanbul, Turkey; 104 Tepecik Children and Research Hospital, Izmir, Turkey; 105 Ege University Faculty of Medicine, Izmir-Bornova, Turkey; 106 Al Jalila Children’s Specialty Hospital, Dubai, United Arab Emirates; 107 Children & Young People’s Kidney Unit, Notthingham, United Kingdom; 108 Children’s Healthcare Pediatric Dialysis Unit, Atlanta, United States; 109 Johns Hopkins Hospital, Baltimore, UK; 110 Children’s Hospital of Alabama, Birmingham, UK; 111 Driscoll Children’s Hospital, Corpus Christi, USA; 112 Texas Children’s Hospital, Houston, USA; 113 University of Iowa Children’s Hospital, Iowa, USA; 114 University of Minnesota Amplatz Children’s Hospital, Minneapolis, USA; 115 The Children’s Hospital of Philadelphia, Philadelphia, USA

## Abstract

While children approaching end-stage kidney disease (ESKD) are considered at risk of uremic anorexia and underweight they are also exposed to the global obesity epidemic. We sought to investigate the variation of nutritional status in children undergoing chronic peritoneal dialysis (CPD) around the globe. The distribution and course of body mass index (BMI) standard deviation score over time was examined prospectively in 1001 children and adolescents from 35 countries starting CPD who were followed in the International Pediatric PD Network (IPPN) Registry. The overall prevalence of underweight, and overweight/obesity at start of CPD was 8.9% and 19.7%, respectively. Underweight was most prevalent in South and Southeast Asia (20%), Central Europe (16.7%) and Turkey (15.2%), whereas overweight and obesity were most common in the Middle East (40%) and the US (33%). BMI SDS at PD initiation was associated positively with current eGFR and gastrostomy feeding prior to PD start. Over the course of PD BMI SDS tended to increase on CPD in underweight and normal weight children, whereas it decreased in initially overweight patients. In infancy, mortality risk was amplified by obesity, whereas in older children mortality was markedly increased in association with underweight. Both underweight and overweight are prevalent in pediatric ESKD, with the prevalence varying across the globe. Late dialysis start is associated with underweight, while enteral feeding can lead to obesity. Nutritional abnormalities tend to attenuate with time on dialysis. Mortality risk appears increased with obesity in infants and with underweight in older children.

## Introduction

The nutritional status is a principal concern when caring for children undergoing chronic peritoneal dialysis (CPD). While early studies revealed providing sufficient nutrition was essential for adequate growth in this population, advances in enteral feeding practices have enabled the elimination of underweight but have not improved linear growth as much as expected^1–3^. Recent concerns have emerged on the potential for adverse effects of excessive caloric intake in patients who receive supplemental feeding^1–3^.

The majority of published studies assessing the nutritional status of dialyzed children were performed at highly specialized pediatric dialysis units in North America and Western Europe. In contrast, on a global scale the risk of nutritional abnormalities in individual regions and countries is likely to be affected by a range of medical and non-medical factors including the patient case-mix regarding age, underlying disease and co-morbidities, national economic strength and healthcare expenditure, cultural acceptability of dietary and feeding prescriptions, availability of special formula diets and enteral feeding equipment, and differences in local, national or regional nutritional recommendations^4^.

The International Pediatric Peritoneal Dialysis Network (IPPN) has been collecting comprehensive clinical and laboratory data in a standardized manner from children undergoing CPD worldwide since 2007. Since these data include detailed anthropometric measures, feeding prescriptions and outcome measures, it provides an opportunity to address the global demographics of nutritional abnormalities in children receiving CPD.

The objective of this study was to examine and follow prospectively the nutritional status of 1,001 children commencing CPD around the globe, analyze factors associated with the nutritional status at the start and during the course of dialysis, and to analyze the impact of nutritional abnormalities on patient survival.

## Methods

### Data collection

The IPPN Registry was established in 2007 and currently collects comprehensive clinical and laboratory information from children undergoing CPD at 95 pediatric dialysis centers in 37 countries around the globe. Patient status is updated every 6 months via an Internet-based web platform (www.pedpd.org). The complete list of data items collected has been published previously^2,4^. Data is automatically checked for plausibility and completeness. Data protection is ensured by pseudonymized data input. The study is performed in accordance with the relevant medical association’s professional codes of conduct with the Declaration of Helsinki from 2008. Approval for the registry project was obtained from The Children’s Mercy Hospital Pediatric Institutional Review Board, Kansas City, USA and local Institutional Review Boards or ethical committees. Informed consent was obtained from the patients and/or their legal guardians as required by local review boards.

### Calculation of BMI SDS and eGFR

Body Mass Index (BMI), i.e. weight/height^2^ (kg/m^2^), was normalized to standard deviation scores (SDS) according to height age, utilizing the WHO (2006) and CDC (2000) standards for children aged younger and older than 5 years, respectively (see www.who.int/childgrowth/en/)^5,6^. Normalization to height age, i.e. the chronological age of a child with the same height growing at the 50^th^ height percentile, was made to adjust for the high prevalence of growth failure in the cohort^7^. BMI SDS values were used to categorize patients into three BMI groups: underweight (<2.5^th^ percentile, i.e. <−2 SDS), normal (2.5^th^ to 85^th^ percentile, i.e. −2 to 1.036 SDS), overweight (>85^th^ −95^th^ percentile, i.e. >1.036 to 1.645 SDS), and obesity (>95^th^ percentile, i.e. >1.645 SDS). The Schwartz bedside formula was used to estimate GFR at initiation of CPD^8^.

### Statistics

Data collection was complete for all observations except residual urine output (7.6% missing data), daily ultrafiltration rate (5.2%), eGFR (1.2%), total PD fluid turnover and dialytic glucose exposure (0.8%), PD modality (0.5%), serum bicarbonate (0.3%), serum albumin (0.2%), and estimated dry weight (0.2%). Multiple imputation by chained equations was conducted to replace these missing values^9^. All analyses were performed using the imputed dataset. Additionally, sensitivity analyses were performed using only cases with complete data sets.

ANOVA or Kruskal-Wallis tests were conducted to compare differences between BMI groups. Differences in proportions were assessed using Chi^2^ tests. Linear mixed modeling was used to identify factors affecting BMI SDS at baseline and during follow-up. The initial cross sectional model included age, sex, eGFR, gross national income (GNI), renal diagnosis, presence of comorbidities, ethnicity, urine output, nutritional support (oral caloric supplements, nasogastric tube (NGT) and gastrostomy feeding), and growth hormone use as independent variables. The region of residence was accounted for as random intercept. For the longitudinal analysis, the change in BMI SDS between two observations, projected to 12 months, was used as the dependent variable and region and patients were used as nested random effects. Potential covariates included in the initial model were age at baseline, sex, presence of comorbidities, renal diagnosis, GNI and the time-varying variables BMI SDS, height SDS, eGFR, % deviation from estimated dry weight, PD modality, duration of PD, serum albumin, serum bicarbonate, total PD fluid volume, urine output, ultrafiltration, growth hormone use, nutritional support, glucose exposure, biocompatible PD fluid use, and amino acid PD fluid use. A stepwise variable selection procedure was applied to identify the relevant covariates for the cross sectional model as well as for the longitudinal model, using p = 0.2 as a cutoff criterion for model entry.

Kaplan–Meier analysis with log-rank testing was used to assess differences in patient survival. Cox proportional hazard modeling with time dependent covariates and interaction term was applied to identify risk factors of death on dialysis.

Data were analyzed using SAS, version 9.3 (SAS Institute, Inc., Cary, NC), and R, version 3.1.1^10^.

## Results

### Study population

All children and adolescents enrolled in the IPPN registry with initiation of CPD between March 2007 and December 2014 were analyzed for this study. Five patients with syndromic and metabolic disorders associated with intrinsic abnormalities of growth and body composition were excluded from the analysis. The final dataset comprised a total of 1,001 incident patients from 85 nephrology centers in 35 countries. Children originated from Western Europe (n = 300), Central Europe (n = 120), Turkey (n = 105), the Middle East (n = 15), China and Hong Kong (n = 77), Korea (n = 24), India and South East Asia (n = 30), New Zealand (n = 18), USA (n = 97), Canada (n = 13) and Latin America (n = 202). One or more comorbidities were reported in 369 patients (35.6%); these included mainly defined syndromic disorders (n = 107), impaired cognitive development (n = 119), cardiac (n = 130) and pulmonary abnormalities (n = 52).

Of the 1,001 patients, 702 (70%) patients had at least two BMI records available. Median follow-up time was 14.5 (IQR 17.8) months. Altogether, the data set contained 2,931 follow-up entries.

### Nutritional status at dialysis initiation

The overall prevalence of underweight, normal weight and overweight/obesity at the start of CPD was 8.9%, 71.4%, and 19.7%, respectively. The detailed patient characteristics according to nutritional status at dialysis entry are shown in Table 1. Overweight/obese children originated from countries with higher GNI per capita, had higher eGFR at CPD initiation and were more growth retarded. Gastrostomy feeding was performed in almost 17% of the overweight children as compared to 8% and 6% in the normal and low BMI groups (p < 0.001 for comparison of gastrostomy feeding between overweight and normal, as well as between overweight and low BMI). Children starting PD with underweight more often received amino acid PD fluid than children without underweight (p = 0.013).

**Table 1:** Patient and treatment baseline characteristics.

	Total population	BMI < 2.5^th^ percentile	BMI 2.5^th^–85^th^ pct	BMI > 85^th^ pct	P
	N = 1001	n = 89	n = 715	n = 197	
Country GNI per capita (1000$)	27.1 ± 13.6	24.0 ± 13.2	27.0 ± 13.4	28.6 ± 14.5	0.03
Age (yrs)	8.5 ± 5.8	7.7 ± 5.9	8.7 ± 5.8	8.1 ± 5.8	0.18
Male gender	550 (55%)	50 (56.2%)	388 (54.3%)	112 (56.9%)	0.79
Renal diagnosis					0.24
CAKUT	424 (42.4%)	44 (49.4%)	291 (40.7%)	89 (45.2%)	
Glomerulopathy	388 (38.8%)	30 (33.7%)	292 (40.8%)	66 (33.5%)	
Other	189 (18.9%)	15 (16.9%)	132 (18.5%)	42 (21.3%)	
Comorbidities	355 (35.5%)	38 (42.7%)	250 (35.0%)	67 (34.0%)	0.32
eGFR at PD start (ml/min/1.73 m^2^)	8.0 (5.3)	7.3 (5.1)	7.9 (5.2)	8.8 (5.6)	0.01
Urine output (L/m^2^/d)	0.61 (0.94)	0.53 (0.85)	0.63 (0.93)	0.56 (1.05)	0.80
PD modality					0.51
CAPD	222 (22.6%)	23 (25.8%)	156 (21.8%)	43 (21.8%)	
APD	759 (77.4%)	60 (67.4%)	547 (76.5%)	152 (77.2%)	
Other	13 (1.3%)	6 (6.7%)	6 (0.8%)	1 (0.5%)	
Biocompatible PD fluid use	428 (42.8%)	33 (37%)	316 (44.2%)	79 (40.1%)	0.31
Height SDS	−1.9 ± 1.7	−1.6 ± 2.1	−1.8 ± 1.6	−2.3 ± 1.8	<0.001
**Nutritional supplementation**					
None	595 (59.4%)	54 (60.7%)	428 (59.9%)	113 (57.4%)	0.01
Oral nutritional supplements	218 (21.8%)	24 (27%)	160 (22.4%)	34 (17.3%)	
NG tube	92 (9.2%)	6 (6.7%)	69 (9.6%)	17 (8.6%)	
Gastrostomy	96 (9.6%)	5 (5.6%)	58 (8.1%)	33 (16.7%)	
Amino acid PD fluid use	12 (1.2%)	4 (4.5%)	5 (0.6%)	3 (1.5%)	0.01
Growth hormone use	64 (6.4%)	3 (3.4%)	50 (7.0%)	11 (5.6%)	0.37
Hemoglobin (g/L)	10.8 ± 1.9	11.0 ± 2.1	10.8 ± 1.9	10.8 ± 1.8	0.61
Serum albumin (g/l)	35.8 ± 6.9	36.2 ± 5.4	35.6 ± 7.2	36.2 ± 6.6	0.512
Blood urea (mg/dl)	96 (63)	93 (64)	97 (63)	93 (62)	0.52
Serum bicarbonate (mM)	23.9 ± 4.3	23.7 ± 4.1	23.9 ± 4.2	24.2 ± 4.7	0.63
Serum phosphorus (mM)	1.8 ± 0.6	1.8 ± 0.6	1.8 ± 0.6	1.7 ± 0.6	0.20

Data are expressed as n (%), median (IQR) or mean ± SD.

**Figure 1:**
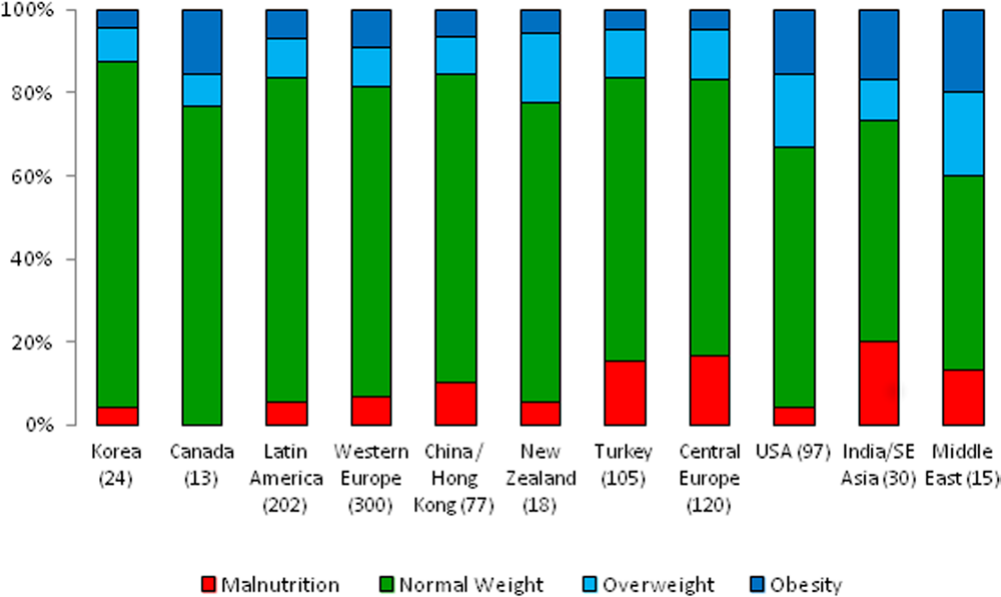
Regional variation of nutritional status at start of CPD, sorted by decreasing fraction of patients with BMI within normal range.

The distribution of underweight, normal weight, and overweight/obese patients according to geographical region is depicted in Fig. 1. The prevalence of nutritional abnormalities varied significantly across world regions. Underweight was most prevalent in South and Southeast Asia (20%), followed by Central Europe (16.7%) and Turkey (15.2%), whereas overweight and obesity were most common in the Middle East (40%) and the US (33%).

The prevalence of underweight was highest in the first year of life (14.2%), decreasing to 6.5%, 9.4% and 7.7% in children aged 1–<6, 6–12 and older than 12 years, respectively (Fig. S1 in supplement). The prevalence of underweight was correlated with the eGFR at initiation of PD, increasing from 5.3% at eGFR 9–12 ml/min/1.73 m^2^ to 11% at eGFR < 6 ml/min/1.73 m^2^ (p = 0.03) (see also Fig. S2). The prevalence of obesity was higher in the younger children than in adolescents, whereas the prevalence of being overweight was largely independent of age (see Fig. S1).

By multivariate analysis, BMI SDS at dialysis initiation was positively predicted by eGFR and the use of gastrostomy feeding and negatively predicted by the presence of comorbidities, whereas age, sex, ethnicity, GNI, renal diagnosis, and growth hormone use were not predictive (Table S1).

### Enteral feeding practices

**Figure 2:**
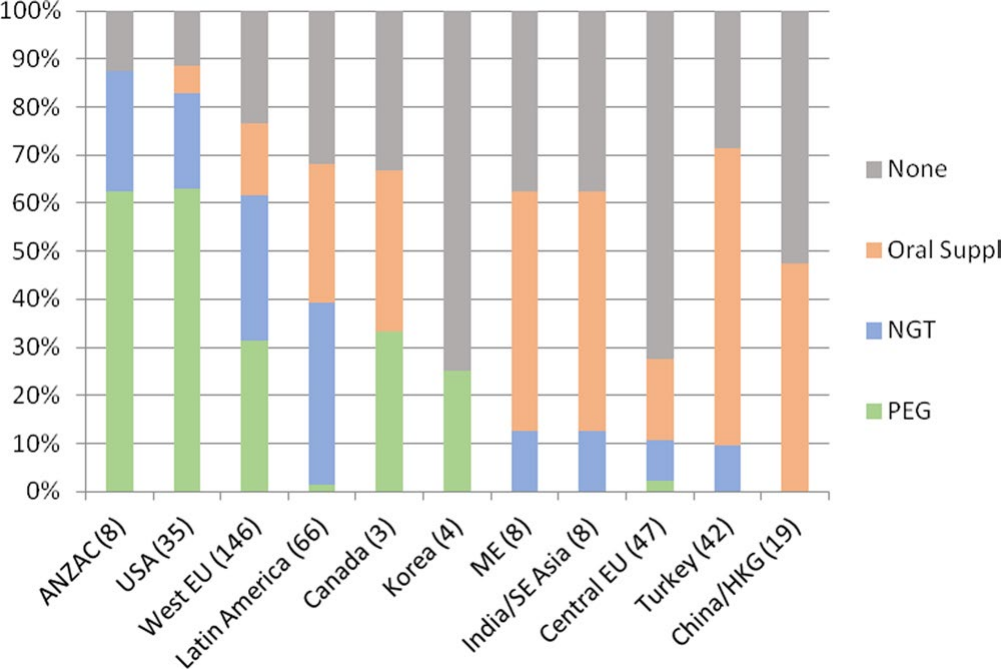
Regional differences in supplementary feeding practices in 386 children <6 years, sorted by increasing fraction of patients without enteral feeding.

To further explore and compare regional characteristics in nutrition management, enteral feeding patterns were investigated. Enteral (NGT or gastrostomy) feeding was used at baseline in 57.4%, 32.9%, 5.6%, and 2.3% of children <1, 1–<6, 6–12 and >12 years, respectively (p < 0.001). The variation of feeding practices varied markedly by region (Fig. 2). Enteral feeding was rarely applied in Central Europe, Turkey, India, South East Asia, and China. Gastrostomy usage was confined to North America, Western Europe, Korea and New Zealand. Among the 386 children <6 years, 166 were followed for at least 12 months on dialysis. The fraction of children with enteral tube feeding was 38% at baseline and 41% at follow up (ns).

### Changes in nutritional status with time on CPD

**Figure 3:**
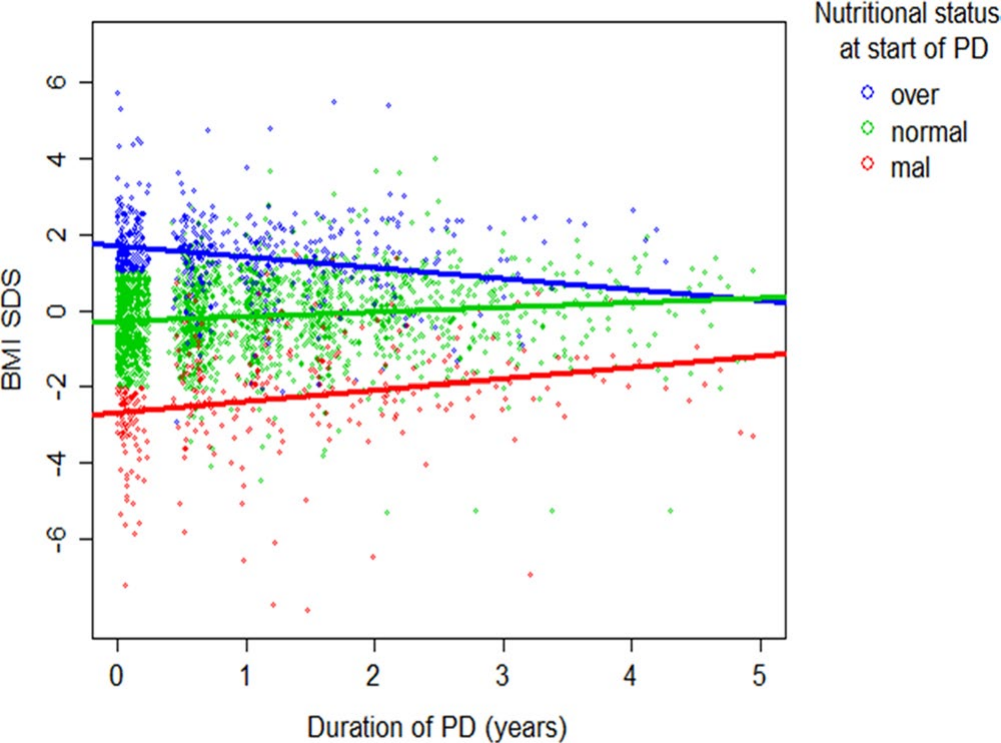
Course of BMI SDS according to nutritional status at start of PD (green: normal BMI, blue: overweight/obese, red: underweight). Regression lines are based on a mixed model predicting BMI SDS from duration of PD, nutritional status at start of PD and their interaction.

Changes in BMI SDS were analyzed using 702 patients with at least two available BMI records. During a median follow-up time of 15 (interquartile range 18) months, BMI SDS tended to increase on CPD in both underweight and normal weight children, whereas it decreased in the overweight patients **(**Fig. 3). Out of 74 underweight children at the start of CPD, 51.4% were non-underweight at last observation; among 125 overweight/obese children at CPD initiation, 36.0% achieved a normal BMI at follow-up.

Gastrostomy feeding was associated with an increase in BMI SDS during follow-up (Table 2). Additional factors independently associated with a positive change in BMI SDS included a lower BMI SDS and higher height SDS, higher serum albumin and the diagnosis of CAKUT. In contrast, greater fluid overload (expressed as % deviation from estimated dry weight) was predictive of a negative change in BMI SDS during follow-up.

**Table 2:** Factors predicting prospective annualized change in BMI SDS.

	Parameter Estimate (SE)		P
Intercept	−0.056	(0.259)	0.89
Duration of PD (years)	−0.060	(0.036)	0.09
**Diagnosis** (***reference: CAKUT***)			
Glomerulopathy	−0.229	(0.089)	0.01
Other	−0.364	(0.110)	0.001
BMI SDS	−0.456	(0.026)	<0.001
Height SDS	0.113	(0.025)	<0.001
% deviation from estimated dry weight	−0.040	(0.014)	0.01
Serum albumin (g/L)	0.015	(0.006)	0.02
**Nutritional supplementation** (***reference: none***)			
Oral	−0.195	(0.090)	0.03
NGT	0.122	(0.133)	0.36
Gastrostomy	0.633	(0.132)	<0.001

Positive change means BMI SDS increase (702 patients, 1930 differences in BMI SDS).

### Nutrition and mortality

**Figure 4:**
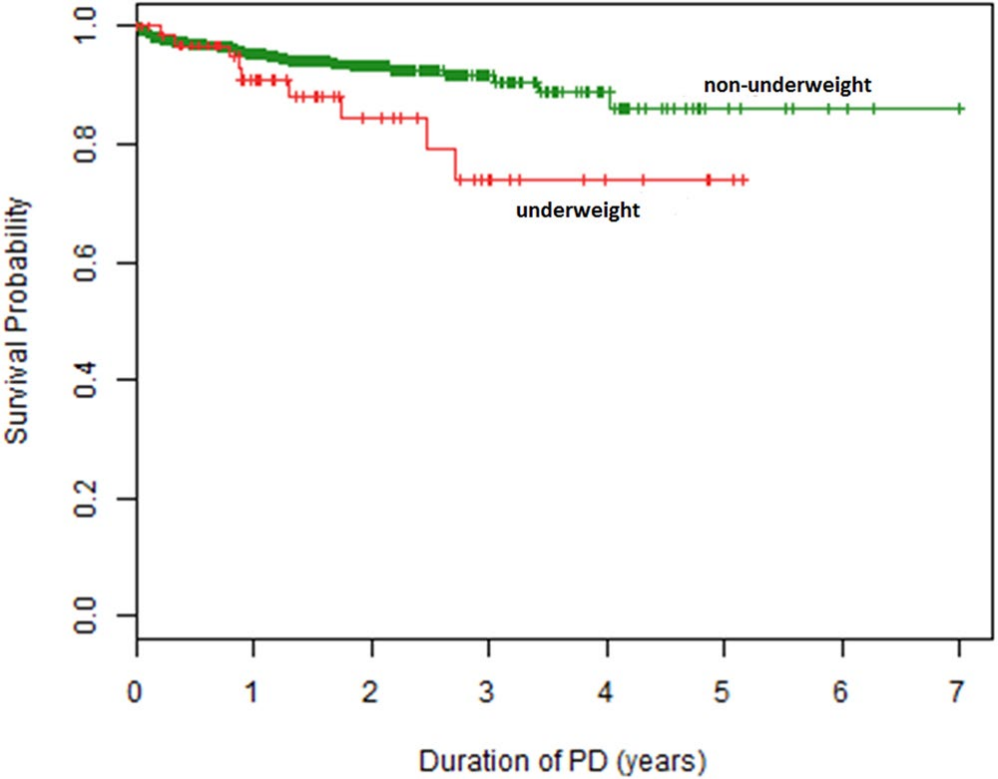
Survival of patients with underweight (BMI < 2.5^th^ percentile) (red) and without underweight (BMI > 2.5^th^ percentile) (green) at last observation (log-rank test: p = 0.03).

**Figure 5:**
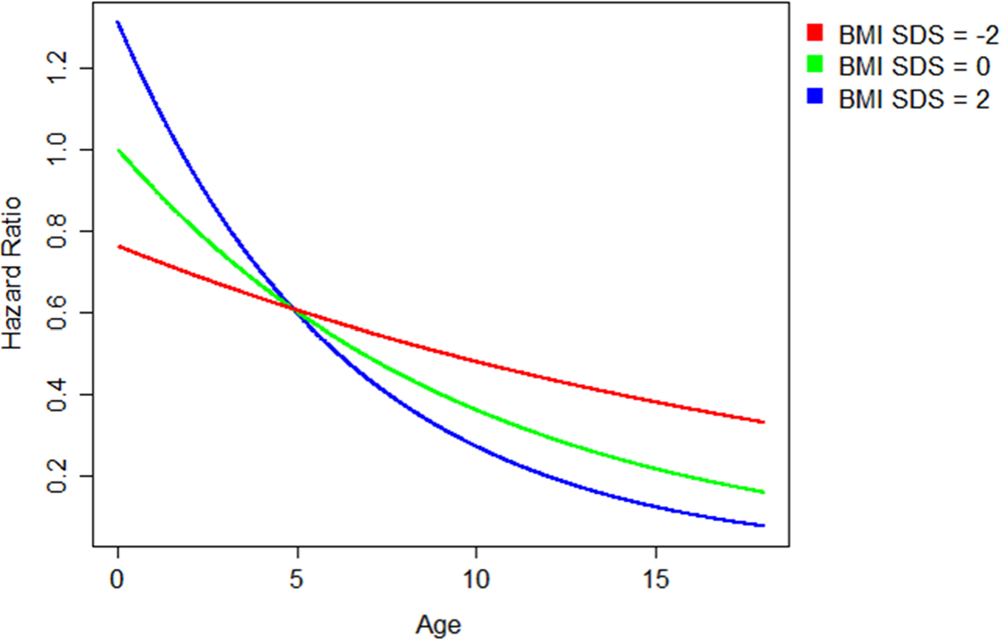
Hazard ratios of death according to age and BMI SDS (reference: age = 0, BMI SDS = 0) for a patient with no comorbidities, based on Cox regression with time dependent variables age, BMI SDS, presence of comorbidities and the interaction of bmi sds and age.

A total of 54 children died during the observation period. The most common causes of death were non-PD related infections (39%), followed by congestive heart failure (17%), PD-related infections (7%) and malignances (7%). The 1-, 2- and 4-year survival rates were 91%, 84%, and 74% in patients who were underweight at last observation, as compared to 95%, 93% and 89% in those with final higher BMI SDS values (p = 0.03) (Fig. 4). Cox proportional hazard analysis identified the presence of comorbidities and younger age as risk factors for death on dialysis (Table 3). Whereas BMI SDS per se was not predictive, the interaction term of BMI SDS and age affected the risk of death at borderline significance (p = 0.06). To further illustrate the interaction of age and BMI SDS with respect to mortality risk on dialysis, we modeled the hazard ratio of death by age for patients with a BMI SDS of −2, 0, and 2 (Fig. 5). While the globally increased risk of death of children younger than 5 years of age is more marked in obese children, at older age underweight children appear at higher risk of death than obese ones.

**Table 3:** Risk factors for death on CPD.

	Estimate	(SE)	Hazard ratio	95% Confidence Interval	P
Comorbidity	0.836	(0.279)	2.307	[1.336, 3.987]	0.001
Age	−0.102	(0.028)	0.903	[0.856, 0.954]	<0.001
BMI SDS	0.136	(0.123)	1.145	[0.900, 1.457]	0.27
BMI SDS * Age	−0.028	(0.015)	0.973	[0.945, 1.002]	0.06

## Discussion

At variance with historical epidemiological studies on nutrition in children with CKD, which focused mainly on uremic underweight, this study highlights a changing trend of the nutritional status in pediatric end stage kidney disease (ESKD). Both underweight and overweight/obesity were common. Using the 2.5^th^ and the 85^th^ BMI percentiles as cutoffs for underweight and overweight/obesity respectively, 28.6% of patients receiving CPD, as compared to an expected 17.5%, exhibited an abnormal body composition.

A relatively low rate of underweight, but an increased prevalence of overweight and obesity was recently also observed in North American CKD cohorts^11,12^ and in European children undergoing dialysis^13^. While it is tempting to merely attribute this trend to the global childhood obesity epidemic, the comprehensive and worldwide data collection in the IPPN Registry allowed us to dissect in detail and on a global scale, the impact of macroeconomic factors, patient characteristics, and pediatric CKD and dialysis management practices on nutritional outcome.

The regional breakdown and comparison with the general childhood population prevalence data revealed that overweight and obesity in the CPD patients was indeed most prevalent in children and adolescents from the US and the Middle East, and comparable to the national prevalence rates in these countries which show the highest prevalence of childhood obesity worldwide^14^. On the other hand, when relating the observed obesity rates to the current prevalences in the general pediatric populations of the respective regions, obesity was slightly less common in patients from Western Europe, but was substantially more frequent in children from South and South East Asia^14^. The latter finding might be explained in part by overrepresentation of families from upper socioeconomic strata to whom chronic dialysis is affordable. Noteworthy also was the finding that whereas gross national income was positively correlated with overweight/obesity prevalence by univariate analysis, it was not included in the multivariate models predicting BMI at initiation of dialysis and during follow-up. This suggests that the observed regional differences can be explained in large part by the medical factors included in the multivariate analysis.

Two factors were consistently associated with BMI SDS at the time of PD initiation: the most current eGFR and the use of gastrostomy feeding prior to PD start. The positive association with eGFR is compatible with the notion that the risk of uremic underweight increases as residual kidney function declines in the late pre-dialytic phase. Centers in countries where late referral to pediatric nephrology care is common may be more likely to start PD in underweight patients with little residual kidney function. In the case of children for whom PD is to be started electively, our findings add a nutritional perspective to the list of factors to be considered when determining the optimal timing of PD initiation.

The most important single factor associated with higher BMI SDS both at PD start and during follow-up was the presence of a gastrostomy. In the multivariate longitudinal analysis, gastrostomy feeding accounted for an increase in BMI by almost two standard deviations per three years of treatment. Accordingly, gastrostomy feeding was almost three times more common among overweight and obese as compared to underweight patients. These findings confirm and extend a previous IPPN study in young infants, which identified PEG feeding as a risk factor for obesity^2^. Since the use of gastrostomy feeding was found to be largely restricted to high-income countries, enteral feeding via gastrostomy appears to be the most important factor underlying the observed link between Gross National Income and higher BMI.

An unexpected observation was the fact that obese - but not underweight - patients were significantly shorter than patients with normal nutritional status at the initiation of renal replacement therapy. This finding, which is in keeping with recent findings in the ESPN/ERA-EDTA registry^13^ and previous reports^15^, may reflect previous frustraneous interventions to correct growth failure by hypercaloric feeding.

An important and reassuring finding from the longitudinal analysis is the observation that nutritional abnormalities, both underweight and overweight/obesity, tend to level off over the course of dialysis. The prevailing BMI SDS was a highly significant inverse predictor of the subsequent change in BMI SDS in the longitudinal model. This ‘funneling’ of the nutritional status is likely to be a consequence of regular nutritional monitoring and dietary advice. Direct effects of dialysis such as control of uremia, correction of acidosis and dialytic glucose resorption probably play a role in the correction of underweight, but these factors were not included as significant predictors of prospective BMI SDS change in the overall model since they are less likely to increase BMI in patients with normal nutritional status.

Remarkably, patients with congenital kidney malformation disorders (CAKUT) were more likely to gain BMI SDS over the course of dialysis than patients with other underlying renal diseases. Since CAKUT is the most common renal diagnosis in infants with ESKD and was slightly more common among initially underweight patients, we speculate that catch-up weight gain due to enteral feeding was more common in this patient group. In addition, as CAKUT patients are often polyuric even at commencement of dialysis, part of the observed BMI gain may be explained by relative fluid gain to the gradual loss of diuresis with time on PD.

The estimated fractional deviation from dry weight was associated with a subsequent negative change in BMI SDS, possibly reflecting the impact of early fluid imbalances (e.g. hypervolemia) and their correction with time on CPD. When patients with substantial fluid overload have their body weight decreased because of improved fluid management, this will be recorded as a negative change in BMI, illustrating the limitation of BMI SDS as a measure of nutritional status.

Nutritional status is a well recognized, major global determinant of mortality in the general adult and pediatric population, as well as in adults and children with ESKD^16–18^. The overall patient survival observed in this global sample of children and adolescents starting CPD (91% at 2 years) was not dissimilar from survival rates noted in North American and European registry cohorts^19–21^. Wong *et al*., in the only other large study examining BMI and mortality in pediatric ESRD, demonstrated a U-shaped association between the risk of death and BMI SDS, with both extremes of BMI associated with increased mortality^19^. An increased death risk was also observed in children with a hematological malignancy who were either underweight or obese^22^. In the present study, underweight patients were twice as likely to die on dialysis as non-underweight children by univariate Kaplan Meier survival analysis, whereas obesity did not affect the risk of death. On the other hand, when accounting for age and the presence of comorbidities in a Cox regression model, although BMI SDS did not predict the risk of death, age and BMI SDS showed an interactive effect; specifically, an increased infant mortality was amplified by obesity, whereas mortality in older children and adolescents was markedly increased by underweight. Hence, our findings are compatible with an age-dependent impact of the extremes of body composition on patient survival. We hypothesize that the morbid obesity generated in a subset of young infants by enteral feeding may, in fact, put these patients at increased risk of death. Possible underlying causes of this risk association might include enhanced cardiovascular risk due to the difficulty of assessing fluid status in obese infants and the susceptibility of obese subjects to severe outcomes following viral respiratory infections^23^.

While the strength of our study relates to the robust set of data available from a large group of pediatric CPD patients, several limitations of our study should be mentioned. Although BMI is a generally accepted method to assess nutritional status in healthy children^24–26^, it is an imperfect measure of body composition. This is particularly true in pediatric dialysis patients as it does not account for abnormalities of fluid status and is impacted by abnormal body height. While the latter issue was accounted for by calculation of BMI SDS for height age^27,28^, a more refined auxological assessment was not possible in this global registry. Another important limitation of our study was the lack of detailed data on dietary prescriptions and caloric intake, which would be difficult to obtain due to complexity of data collection in such a large and diverse cohort of patients.

In summary, this longitudinal assessment of 1001 children from 35 countries commencing CPD demonstrates that both underweight and obesity are observed at increased frequency in pediatric ESRD. The prevalence of both abnormalities varies substantially across the globe. Delayed start of dialysis is a risk factor for underweight whereas enteral tube feeding, while protecting from underweight, increases the risk of developing obesity. Nutritional abnormalities tend to attenuate with time on dialysis.

## Supplementary information

Dataset1
