# Supplementary material for: Global Variation of Nutritional Status in Children Undergoing Chronic Peritoneal Dialysis: A Longitudinal Study of the International Pediatric Peritoneal Dialysis Network
Source: Sci Rep. 2019 Mar 20;9:4886. doi: 10.1038/s41598-018-36975-z (PMC6426856; doi:10.1038/s41598-018-36975-z)
Supplement: Supplementary file 1 — Dataset1 [file 41598_2018_36975_MOESM1_ESM.docx]

**Global Variation of Nutritional Status in Children Undergoing Chronic Peritoneal Dialysis: A Longitudinal Study of the International Pediatric Peritoneal Dialysis Network**

Franz Schaefer^1^, Laura Benner^2^, Dagmara Borzych-Duzalka^3^, Joshua Zaritsky^4^, Hong Xu^5^, Lesley Rees^6^, Zenaida L. Antonio^7^, Erkin Serdaroglu^8^, Nakysa Hooman^9^ , Hiren Patel^10^, Lale Sever^11^, Karel Vondrak^12^, Joseph Flynn^13^, Anabella Rébori ^14^, William Wong^15^, Tuula Hölttä^16^, Zeynep Yuruk Yildirim^17^, Bruno Ranchin^18^, Ryszard Grenda^19^, Sara Testa^20^ , Dorota Drozdz^21^, Attila J Szabo^22^, Loai Eid^23^ , Biswanath Basu^24^, Renata Vitkevic^25^, Cynthia Wong^26^, Stephen J Pottoore^27^ , Dominik Müller^28^, Ruhan Dusunsel^29^, Claudia Gonzalez Celedon^30^, Marc Fila^31^ , Lisa Sartz^32^, Anja Sander^2^, Bradley A Warady^33^, for the International Pediatric Peritoneal Dialysis Network (IPPN) Registry

**Table S-1**. Multivariate analysis of factors predicting BMI SDS at start of CPD.

|  | **Estimate (SE)** | | P |
| --- | --- | --- | --- |
| Intercept | -0.284 | (0.130) | 0.03 |
| eGFR | 0.020 | (0.009) | 0.04 |
| Comorbidities | -0.156 | (0.101) | 0.12 |
| Nutritional supplementation *(reference = none)*  Oral  NGT  Gastrostomy | -0.180  0.156  0.446 | (0.117)  (0.169)  (0.169) | 0.12  0.36  0.01 |

**Global Variation of Nutritional Status in Children Undergoing Chronic Peritoneal Dialysis: A Longitudinal Study of the International Pediatric Peritoneal Dialysis Network**

Franz Schaefer^1^, Laura Benner^2^, Dagmara Borzych-Duzalka^3^, Joshua Zaritsky^4^, Hong Xu^5^, Lesley Rees^6^, Zenaida L. Antonio^7^, Erkin Serdaroglu^8^, Nakysa Hooman^9^ , Hiren Patel^10^, Lale Sever^11^, Karel Vondrak^12^, Joseph Flynn^13^, Anabella Rébori ^14^, William Wong^15^, Tuula Hölttä^16^, Zeynep Yuruk Yildirim^17^, Bruno Ranchin^18^, Ryszard Grenda^19^, Sara Testa^20^ , Dorota Drozdz^21^, Attila J Szabo^22^, Loai Eid^23^ , Biswanath Basu^24^, Renata Vitkevic^25^, Cynthia Wong^26^, Stephen J Pottoore^27^ , Dominik Müller^28^, Ruhan Dusunsel^29^, Claudia Gonzalez Celedon^30^, Marc Fila^31^ , Lisa Sartz^32^, Anja Sander^2^, Bradley A Warady^33^, for the International Pediatric Peritoneal Dialysis Network (IPPN) Registry

**Fig.S-1** Variation of nutritional status at start of CPD by age. Red, green, light blue and dark blue bars depict fraction of patients with BMI < 2.5^th^, 2.5^th^-85^th^, 85^th^-95^th^, and >95^th^ percentile for height age.


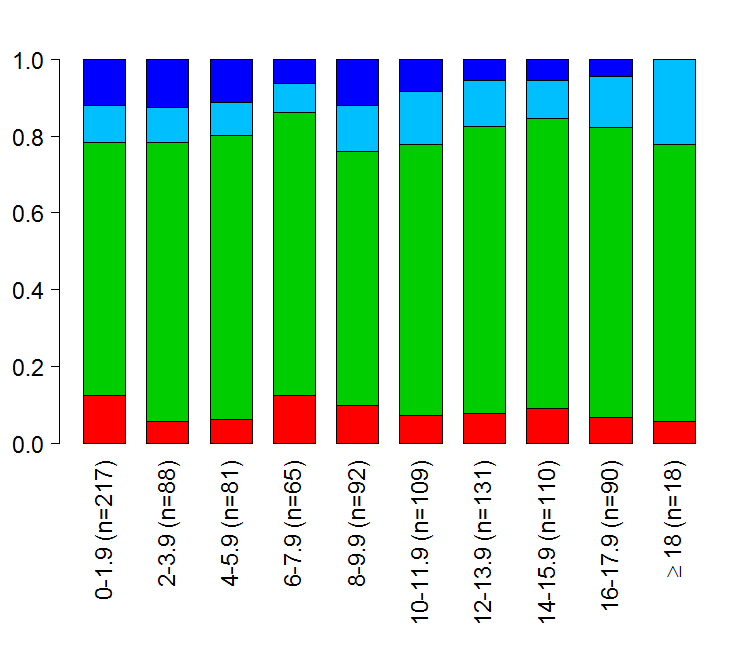


**Global Variation of Nutritional Status in Children Undergoing Chronic Peritoneal Dialysis: A Longitudinal Study of the International Pediatric Peritoneal Dialysis Network**

Franz Schaefer^1^, Laura Benner^2^, Dagmara Borzych-Duzalka^3^, Joshua Zaritsky^4^, Hong Xu^5^, Lesley Rees^6^, Zenaida L. Antonio^7^, Erkin Serdaroglu^8^, Nakysa Hooman^9^ , Hiren Patel^10^, Lale Sever^11^, Karel Vondrak^12^, Joseph Flynn^13^, Anabella Rébori ^14^, William Wong^15^, Tuula Hölttä^16^, Zeynep Yuruk Yildirim^17^, Bruno Ranchin^18^, Ryszard Grenda^19^, Sara Testa^20^ , Dorota Drozdz^21^, Attila J Szabo^22^, Loai Eid^23^ , Biswanath Basu^24^, Renata Vitkevic^25^, Cynthia Wong^26^, Stephen J Pottoore^27^ , Dominik Müller^28^, Ruhan Dusunsel^29^, Claudia Gonzalez Celedon^30^, Marc Fila^31^ , Lisa Sartz^32^, Anja Sander^2^, Bradley A Warady^33^, for the International Pediatric Peritoneal Dialysis Network (IPPN) Registry

**Fig. S-2** Mean BMI SDS at first observation according to eGFR at initiation of CPD.
